# Association between different MAP levels and 30-day mortality in sepsis patients: a propensity-score-matched, retrospective cohort study

**DOI:** 10.1186/s12871-023-02047-7

**Published:** 2023-04-06

**Authors:** Xiaoxin Zhong, Haifeng Li, Qian Chen, Peng Hao, Tong Chen, Hantao Mai, Kelin Zhang, Guifang Zhong, Ruilian Guo, Huihua Cheng, Benhua Jiang, Sicong Zhu, Suyuan Zhuang, Haoran Li, Yantao Chen, Qing He

**Affiliations:** 1 Department of Surgical Intensive Care Unit, Sun Yat-sen Memorial Hospital, Sun Yat-sen University, No.107, Yanjiang West Road, Yuexiu District, Guangzhou, 510120 China; 2 Department of Pediatric, The Third Affiliated Hospital of Sun Yat-sen University, Sun Yat-sen University, No.600, Shipai Street, Tianhe District, Guangzhou, 510000 China; 3 Department of Orthopedics, Sun Yat-sen Memorial Hospital, Sun Yat-sen University, No.107, Yanjiang West Road, Yuexiu District, Guangzhou, 510120 China

**Keywords:** Sepsis, Mean arterial pressure, Mortality, Retrospective study

## Abstract

**Background:**

Sepsis is a life-threatening organ dysfunction caused by the infection-related host response disorder. Adequate mean arterial pressure is an important prerequisite of tissue and organ perfusion, which runs through the treatment of sepsis patients, and an appropriate mean arterial pressure titration in the early-stage correlates to the positive outcome of the treatment. Therefore, in the present study, we aimed to elucidate the relationship between early mean arterial pressure levels and short-term mortality in sepsis patients.

**Methods:**

We included all suspected sepsis patients from MIMIC-III database with average mean arterial pressure ≥ 60 mmHg on the first day of intensive care unit stay. Those patients were then divided into a permissive low-mean arterial pressure group (60–65 mmHg) and a high-mean arterial pressure group (> 65 mmHg). Multivariate Cox regression analysis was conducted to analyze the relationship between MAP level and 30-day, 60-day, and 100-day mortality of suspected sepsis patients in the two groups. Propensity score matching, inverse probability of treatment weighing, standardized mortality ratio weighting, PA weighting, overlap weighting, and doubly robust analysis were used to verify our results.

**Results:**

A total of 14,031 suspected sepsis patients were eligible for inclusion in our study, among which 1305 (9.3%) had an average first-day mean arterial pressure of 60–65 mmHg, and the remaining 12,726 patients had an average first-day mean arterial pressure of more than 65 mmHg. The risk of 30-day mortality was reduced in the high mean arterial pressure group compared with the permissive low-mean arterial pressure group (HR 0.67 (95% CI 0.60–0.75; p < 0.001)). The higher mean arterial pressure was also associated with lower 60-day and 100-day in-hospital mortality as well as with shorter duration of intensive care unit stay. Patients in the high-mean arterial pressure group also had more urine output on the first and second days of intensive care unit admission.

**Conclusions:**

After risk adjustment, the initial mean arterial pressure of above 65 mmHg was associated with reduced short-term mortality, shorter intensive care unit stay, and higher urine volume in the first two days among patients with sepsis.

**Supplementary Information:**

The online version contains supplementary material available at 10.1186/s12871-023-02047-7.

## Background

Sepsis is now the leading cause of death from infection in the world with nearly 20 million people suffering from severe sepsis every year [1]. More than 10,000 people die of sepsis-related complications every day [2]. According to the data released by the Global Sepsis Alliance, the number of deaths caused by sepsis exceeds the combined number of deaths related to prostate cancer, breast cancer, and AIDS [3].

The urgency of decisive progress in the treatment of sepsis is increasing. The morbidity of sepsis in developed countries increased at an annual rate of about 10% in the past 10 years [4] and is becoming an accumulative burden for the whole world. In developing countries, the mortality of sepsis remained high for a long time as a result of poverty, hunger, and lack of effective management [5, 6], and the fact that complete epidemiological surveys are generally rare. These alarming facts should serve as a wake-up call to motivate research into improved sepsis treatment strategies.

Hemodynamic management runs throughout the clinical therapy of patients with sepsis [7, 8], and is also critical for stabilizing vital signs at early stage, delaying disease progression and avoiding further tissue and organ failure [9]. However, the range of initial Mean Arterial Pressure (MAP) titration for patients with sepsis remains controversial. An inappropriately low initial MAP inevitably results in hypoperfusion in tissues and organs [10], but if MAP is kept at a high level, the dose of vasoactive drugs needs to be quite high, and the body may suffer from future re-injury. Therefore, the question of how to set an appropriate level of initial MAP that can be of maximum benefit to patients is under fierce discussion. A recent multi-center randomized controlled study (RCT) found no significant difference in mortality between patients with permissive hypotension (MAP 60–65 mmHg) and those with conventional therapy in vasodilatory shock patients (41.0% vs. 43.8%) [11]. Another multi-center RCT found no difference in 28-day mortality between shock patients in the high and low MAP groups [12]. The 2021 international guidelines for the management of sepsis and septic shock set the initial MAP target for sepsis patients as 65 mmHg instead of the higher values [13]. However, in all the studies mentioned above, the actual blood pressure of the permissive low-MAP group was significantly higher than the pre-set blood pressure target, which inevitably influenced the accuracy of the conclusions.

Our study, based on a large public clinical database called the MIMIC-III, assessed the average level of first-day MAP in suspected sepsis patients, aiming to investigate the relationship between different MAP levels and short-term mortality. Propensity Score Matching (PSM) was undertaken to diminish the influence of confounding factors on the results such as age, gender, underlying disease, and initial severity of disease. Meanwhile, our study utilized the actual MAP values of critically ill patients, which cannot be completely controlled in the preset target range in traditional RCT studies.

## Methods

### Data source

The data were obtained from the Medical Information Mart for Intensive Care III (MIMIC-III) database, which covers detailed clinical information of 38,597 adult patients admitted to Beth Israel Deaconess Medical Center, Massachusetts, USA from 2001 to 2012 [14]. The MIMIC-III dataset is freely available after passing an official assessment. One author has passed a series of courses offered by the National Institutes of Health and has gained certification to use the relevant information in the MIMIC-III database (certification number: 40,489,150). Our study conformed to the Reporting of Studies Conducted using observation Routinely Collected Health Data (RECORD) statement [15]. The MIMIC-III database has been approved by the Institutional Review Board (IRB) of MIT and BIDMC, both of which exempt MIMIC-III database related research from the requirement of informed consent, so this study did not require approval by the ethics committee of our hospital.

### Selection of participants

We searched available records in the MIMIC-III database from June 2001 to October 2012 and included all suspected sepsis patients who met the diagnostic criteria of suspected infection with a Sequential Organ Failure Assessment (SOFA) score ≥ 2 according to the third international consensus definition of sepsis and septic shock (Sepsis-3.0) [16]. The patients who met the following criteria were excluded: ① patients who were not admitted to ICU for the first time; ② patients younger than 18 years old; ③ patients with MAP average less than 60 mmHg on the first day of ICU admission; ④ patients with inaccurate recorded time of death.

### Study variables and outcomes

Baseline characteristics and measurement of interest in the MIMIC-III database were extracted using a common structured query language (SQL), including demographics (gender, age, weight), ICU admission category, critical illness score, vital signs, comorbidity, extracorporeal life support(ELS) and laboratory test results (first day of admission). ICU admission categories included medical ICU, surgery/trauma ICU, and cardiac surgery ICU. Critical illness scores included Sequential Organ Failure Assessment(SOFA) score, Simplified Acute Physiology Score II(SAPS II) score, and Acute Severity of Illness Score(OASIS) score. Vital signs included heart rate, mean arterial pressure, respiratory rate, temperature, and oxygen saturation. Comorbidities included congestive heart failure(CHF), cardiac arrhythmia, hypertension, stroke, chronic obstructive pulmonary disease(COPD), diabetes mellitus, renal failure, liver disease, malignancy, and coagulopathy. Extracorporeal life support(ELS) included renal replacement therapy(RRT), mechanical ventilation(MV), vasoactive drugs, and use of vasopressors. Laboratory tests included white blood cell count(WBC), hemoglobin, platelet count(PLT), hematocrit, international normalized ratio(INR), prothrombin time(PT), activated partial thromboplastin time(APTT), blood urea nitrogen(BUN), creatinine, serum potassium, serum sodium, bicarbonate, pH, oxygen partial pressure(PO_2_), carbon dioxide partial pressure(PCO_2_), lactic acid, and anion gap.

The outcomes were also extracted, including 30-day mortality, 60-day mortality, 100-day mortality as well as in-hospital mortality, ICU-free days in 28 days, AKI within 48 h, AKI within 7 days, urine-output on day 1, urine-output on day 2, urine-output on day 3. The primary outcomes were 30-day mortality, 60-day mortality, 100-day mortality while in-hospital mortality, ICU-free days in 28 days, AKI within 48 h, AKI within 7 days, urine-output on day 1, urine-output in day 2, urine-output on day 3 was defined as the secondary outcome.

### Analysis

Baseline characteristics and measurement of interest conforming to normal distribution were expressed as the mean ± standard deviation ($\overline{x}+s$), and those variables that did not conform to a normal distribution were expressed as the median (M) and interquartile range (IQR). The chi-square test, one-way ANOVA, and Kruskal-Wallis test were utilized for the comparison of categorical, normally distributed, and non-normally distributed continuous variables, respectively. Multivariate COX regression was carried out to observe the relationship between patients with different MAP levels and the main outcomes in the two groups.

To avoid bias generated by missing data, the analysis of the primary outcome was re-performed after multiple imputations. To minimize the potential bias of treatment allocation and confounders, COX regression and Propensity Score Matching (PSM) [17, 18] were also utilized to adjust the covariates and confirm the robustness of our findings. One-to-one nearest neighbor matching was applied with a caliper width of 0.2 [19]. All variables were adjusted by PSM. The Standardized Mean Differences (SMD) were computed to examine the effectiveness of PSM. A threshold of less than 0.1 was considered acceptable. In the matched cohort, we used a 2-sided t-test to compare length of ICU-free stay in 28 days and urine output on day 1, 2, 3. We used the chi-square test to compare in-hospital mortality, AKI within 48 h, and AKI within 7 days. We applied the Kaplan-Meier and log-rank analyses to determine 30-day, 60-day, and 100-day survival curves.

Using the estimated propensity scores as weights, an Inverse Probability of Treatment Weighting (IPTW) [20] model was used to generate a weighted cohort. Univariable Cox proportional hazards regression was then performed to adjust the propensity score. Standardized Mortality Ratio Weighting (SMRW), PA weighting (PA) and Overlap Weighting (OW) were also conducted to adjust the covariates to validate the robustness of our results. We also performed univariable Cox proportional hazards regression model with the robust variance estimator to calculate the hazard ratio (HR) for mortality [21].

All analyses were performed using the statistical software package R version 3.4.3 (R Foundation for Statistical Computing, Vienna, Austria). A threshold of p < 0.05 (two-sided) was considered statistically significant.

## Results

### Study population and baseline characteristics

**Fig. 1:**
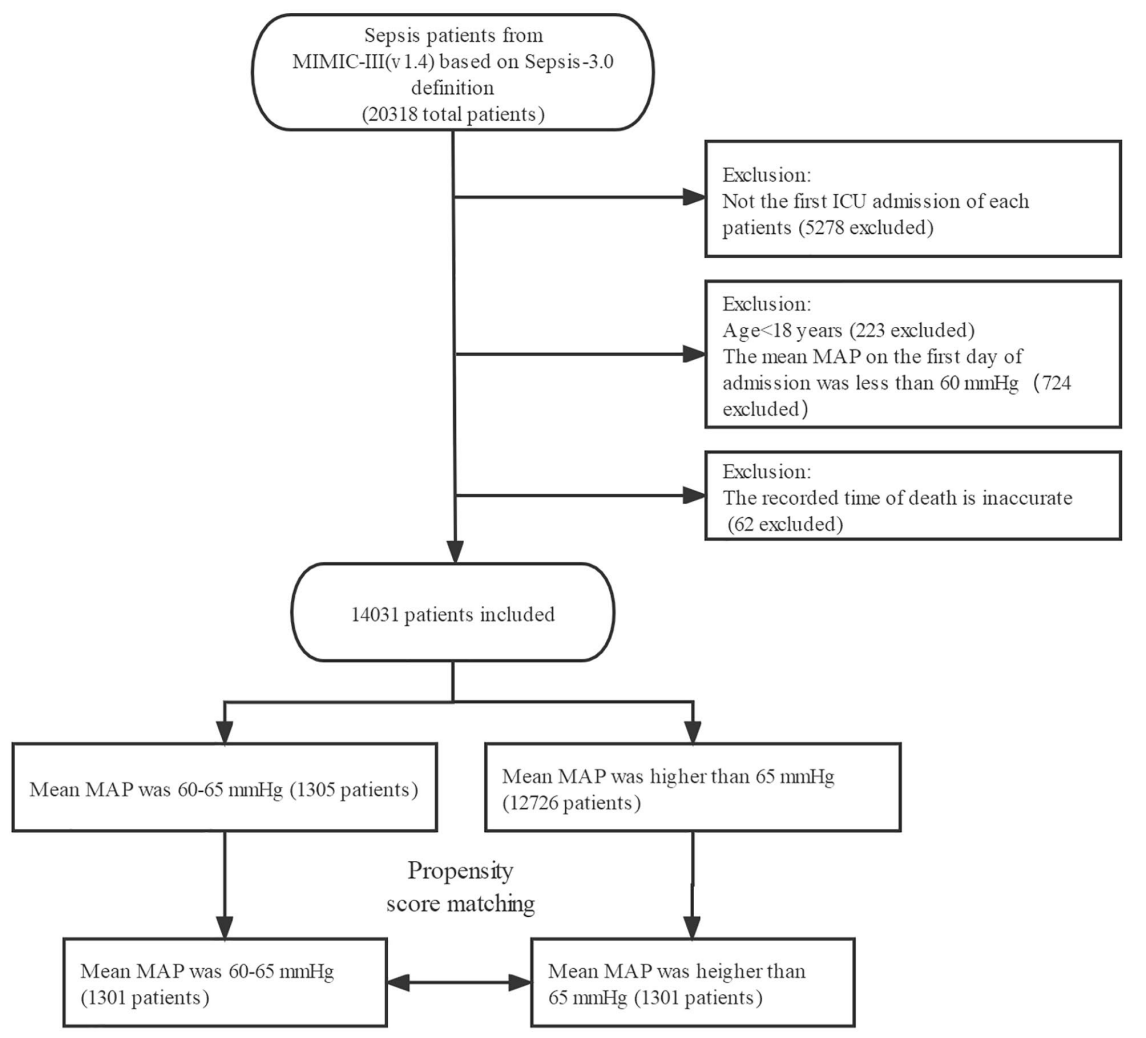
Flow diagram for data inclusion in the present study

With data of 20,318 suspected sepsis patients in the MIMIC-III database reviewed, a total of 14,031 patients were included in the original cohort after applying the exclusion criteria, including 1305 patients with MAP from 60 to 65 mmHg on the first day. After propensity score matching, 1301 patients in both the permissive low-MAP group and the high-MAP group were successfully matched and included in the current study. The flow chart of screening and matching is shown in Fig. 1. Demographic characteristics (gender, age, body weight), category of initial admission to ICU, critical illness score, vital signs, comorbidities, extracorporeal life support, and laboratory test results were presented in the form of comparison of pre-matching and after-matching, as shown in Table 1. Visualized comparison curves of weighted data before and after matching are shown in Figures S1, S2. The absence of all baseline data prior to matching is shown in additional Table S1. The comparison of critical illness scores between the two groups before and after matching can be seen in Figure S3. Univariate analysis was used to investigate the relationship between all covariates of baseline data and 30-day mortality, as presented in Table S2. The distribution of MAP before and after PSM is compared in Table S3.

**Table 1:** Comparisons of baseline characteristics between the original cohort and matched cohort

Characteristic	Original cohort				Matched cohort		
	MAP (60–65 mmHg)	MAP (> 65 mmHg)	SMD		MAP (60–65 mmHg)	MAP (> 65 mmHg)	SMD
	(*n* = 1305)	(*n* = 12,726)			(*n* = 1301)	(*n* = 1301)	
female,n (%)	692 (53)	5805 (45.6)	0.149		689 (53)	716 (55)	0.042
Age (years)	72.1 ± 14.8	66.5 ± 16.4	0.359		72.0 ± 14.8	71.4 ± 14.0	0.047
Service unit, n (%)			0.257				0.031
MICU	782 (59.9)	6235 (49)			779 (59.9)	762 (58.6)	
SICU/TSICU	225 (17.2)	3426 (26.9)			225 (17.3)	239 (18.4)	
CCU/CSRU	298 (22.8)	3065 (24.1)			297 (22.8)	300 (23.1)	
Weight (kg)	78.1 ± 23.9	81.1 ± 25.3	0.123		78.1 ± 23.9	77.5 ± 22.5	0.025
Vital signs							
Heart rate (bpm)	85.8 ± 16.5	88.6 ± 16.4	0.176		85.7 ± 16.5	86.0 ± 16.5	0.013
Respiratory ate (bpm)	20.2 ± 4.5	19.8 ± 4.4	0.094		20.2 ± 4.5	20.5 ± 4.6	0.05
Temperature (℃)	36.8 ± 0.7	36.9 ± 0.7	0.168		36.8 ± 0.7	36.8 ± 0.7	0.018
SpO_2_ (%)	96.6 ± 3.8	97.0 ± 2.6	0.138		96.6 ± 3.8	96.5 ± 3.8	0.027
Severity of illness							
Sofa	5.0 (4.0, 8.0)	5.0 (3.0, 7.0)	0.262		5.0 (4.0, 8.0)	5.0 (4.0, 8.0)	0.018
SapsII	45.6 ± 14.4	40.1 ± 13.9	0.384		45.5 ± 14.2	45.3 ± 15.4	0.013
Oasis	35.5 ± 8.7	34.5 ± 8.7	0.12		35.5 ± 8.7	35.4 ± 9.4	0.007
Comorbidities, n (%)							
CHF	379 (29)	2934 (23.1)	0.137		377 (29)	379 (29.1)	0.003
Cardiac arrhythmias	388 (29.7)	3062 (24.1)	0.128		387 (29.7)	375 (28.8)	0.02
Hypertension	234 (17.9)	1761 (13.8)	0.112		233 (17.9)	232 (17.8)	0.002
Stroke	38 (2.9)	493 (3.9)	0.053		38 (2.9)	47 (3.6)	0.039
COPD	289 (22.1)	2725 (21.4)	0.018		287 (22.1)	276 (21.2)	0.021
Diabetes mellitus	403 (30.9)	3574 (28.1)	0.061		403 (31)	410 (31.5)	0.012
Renal failure	303 (23.2)	2125 (16.7)	0.164		302 (23.2)	305 (23.4)	0.005
Liver disease	138 (10.6)	1016 (8)	0.089		137 (10.5)	120 (9.2)	0.044
Malignancy	185 (14.2)	1316 (10.3)	0.117		181 (13.9)	158 (12.1)	0.053
Coagulopathy	305 (23.4)	2182 (17.1)	0.155		304 (23.4)	305 (23.4)	0.002
ELS, n (%)							
RRT use (hours)	465 (35.6)	6556 (51.5)	0.325		465 (35.7)	448 (34.4)	0.027
MV use (hours)	90 (6.9)	606 (4.8)	0.091		90 (6.9)	86 (6.6)	0.012
Vasopressor use (hours)	247 (18.9)	2121 (16.7)	0.059		246 (18.9)	235 (18.1)	0.022
Laboratory tests							
WBC (10^9^/L)	13.2 (9.1, 18.7)	13.1 (9.3, 18.2)	0.029		13.2 (9.1, 18.7)	12.9 (9.1, 18.3)	0.004
Hemoglobin(g/dL)	9.4 ± 1.8	9.9 ± 2.1	0.283		9.4 ± 1.8	9.4 ± 2.0	0.008
Platelet (10^9^/L)	183.0 (112.0, 261.0)	184.0 (121.0, 259.0)	0.022		183.0 (112.0, 261.0)	180.0 (118.0, 258.0)	0.001
Hematocrit (%)	28.1 ± 5.4	29.4 ± 6.1	0.227		28.1 ± 5.4	28.1 ± 5.9	0.001
INR	1.5 (1.2, 2.0)	1.3 (1.2, 1.7)	0.151		1.5 (1.2, 2.0)	1.4 (1.2, 1.9)	0.009
PT (seconds)	15.8 (13.9, 19.6)	14.8 (13.4, 17.4)	0.198		15.8 (13.9, 19.6)	15.3 (13.7, 19.3)	0.008
APTT (seconds)	36.5 (29.6, 52.0)	33.3 (27.9, 45.3)	0.14		36.5 (29.6, 52.0)	35.7 (28.8, 52.5)	0.003
Bun (mg/dL)	34.0 (21.0, 54.0)	25.0 (16.0, 41.0)	0.333		34.0 (21.0, 54.0)	33.0 (20.0, 53.0)	0.039
Creatinine (mg/dL)	1.5 (1.0, 2.5)	1.2 (0.8, 1.9)	0.209		1.5 (1.0, 2.5)	1.5 (1.0, 2.6)	0.004
Potassium (mmol/L)	3.8 (3.4, 4.3)	3.7 (3.4, 4.1)	0.192		3.8 (3.4, 4.3)	3.8 (3.4, 4.2)	0.016
Sodium (mmol/L)	135.5 ± 6.3	136.4 ± 5.6	0.159		135.5 ± 6.3	135.5 ± 7.2	0.001
Bicarbonate (mmol/L)	20.9 ± 5.5	21.9 ± 5.3	0.192		20.9 ± 5.5	20.8 ± 5.5	0.012
pH	7.4 ± 0.1	7.4 ± 0.1	0.102		7.4 ± 0.1	7.4 ± 0.1	0.012
PO_2_ (mmHg)	102.0 (70.0, 176.0)	116.0 (77.0, 209.0)	0.158		103.0 (70.0, 176.0)	99.0 (70.0, 163.0)	0.039
PCO_2_ (mmHg)	41.2 ± 12.0	41.9 ± 12.2	0.059		41.2 ± 12.0	40.7 ± 11.9	0.043
Lac (mmol/L)	2.2 (1.5, 3.5)	2.2 (1.4, 3.5)	0.056		2.2 (1.5, 3.5)	2.2 (1.4, 3.7)	0.028
Anion gap (mmol/L)	16.0 (14.0, 20.0)	16.0 (13.0, 19.0)	0.131		16.0 (14.0, 20.0)	16.0 (14.0, 20.0)	0.026

*MAP* mean arterial pressure, *MICU* medical intensive care, *SICU* surgical intensive care unit, *TSICU* trauma surgical intensive care unit, *CCU* coronary care unit, *CSRU* cardiac surgery unit, *SOFA* Sequential Organ Failure Assessment, *SAPS II* Simplified Acute Physiology Score II, *Oasis* Oxford Acute Severity of Illness Score, *ELS* extracorporeal life support *MV* mechanical ventilation, *RRT* renal replacement therapy, *CHF* congestive heart failure, *COPD* chronic obstructive pulmonary disease, *WBC* white blood cell, *INR* international normalized ratio, *PT* prothrombin time, *APTT* activated partial thromboplastin time, *BUN* blood urea nitrogen, *PO*_*2*_ partial pressure of oxygen, *PCO*_*2*_ partial pressure of carbon dioxide, *Lac* lactic acid;

**Fig. 2:**
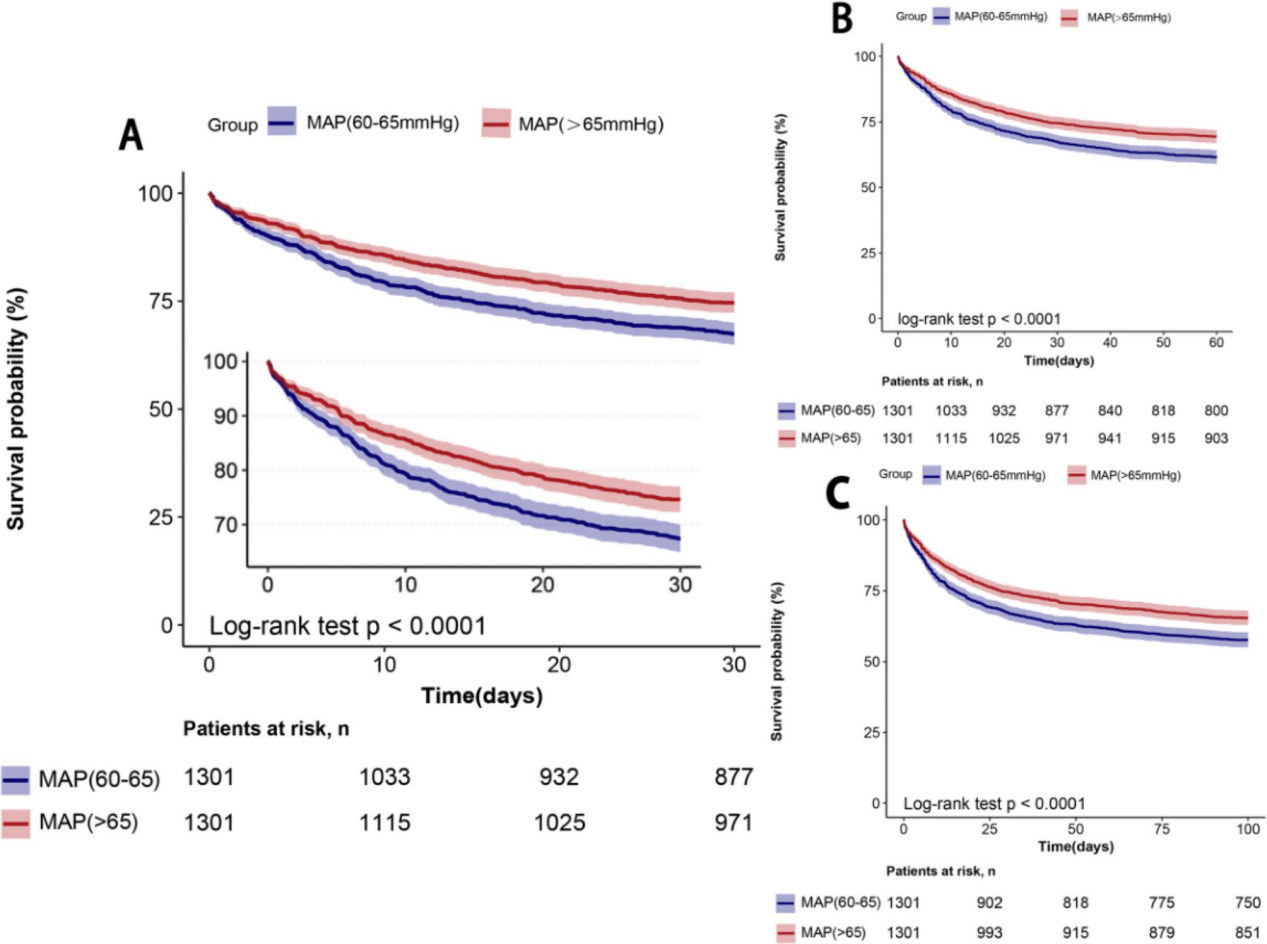
Kaplan-Meier Curves for 30-day (A), 60-day (B),100-day (C) survival probability comparing patients with permissive low-MAP and high-MAP

### Primary outcome

After conducting PSM, the 30-day mortality of the permissive low-MAP group and the high-MAP group were 32.6% (424/1301) and 25.4% (330/1301), respectively, and the 60-day mortality was 38.5% (501/1301) and 30.6% (398/1301), respectively. The 100-day mortality of sepsis patients was 42.4% (551/1301) and 34.6% (450/1301), respectively. Kaplan-Meier curves depicted that the mortality of the high-MAP group at 30, 60 and 100 days was lower than that of the permissive low-MAP group (Log-rank test: p < 0.0001, Fig. 2A and B C, Table 2). Additionally, the Kaplan-Meier curves of 30-day, 60-day, and 100-day mortality in the two groups before and after matching are shown in Figures S4, S5, and S6.

**Table 2:** Clinical outcomes analysis with propensity score matched cohorts

Outcomes	Total (*n* = 2602)	MAP(60–65 mmHg)	MAP(> 65 mmHg)	*P* value
		(*n* = 1301)	(*n* = 1301)	
Primary outcome				
30-day mortality	754 (29.0)	424 (32.6)	330 (25.4)	< 0.001
60-day mortality	899 (34.6)	501 (38.5)	398 (30.6)	< 0.001
100-day mortality	1001 (38.5)	551 (42.4)	450 (34.6)	< 0.001
Secondary outcome				
In-hospital mortality	642 (24.7)	359 (27.6)	283 (21.8)	< 0.001
ICU-free day in 28days	25.1 (7.1, 28.0)	24.6 (3.8, 27.9)	25.7 (9.6, 28.1)	< 0.001
AKI within 48 h, n (%)	1580 (60.7)	807 (62)	773 (59.4)	0.185
AKI within 7 day, n (%)	2096 (80.6)	1049 (80.6)	1047 (80.5)	0.96
Urine-output on day 1 (ml)	1217.0 (650.0, 2010.0)	1150.0 (640.0, 1880.0)	1300.0 (665.0, 2170.0)	0.007
Urine-output on day 2 (ml)	1132.0 (526.5, 1936.0)	1059.0 (485.0, 1872.0)	1212.0 (579.2, 2026.0)	0.013
Urine-output on day 3 (ml)	1179.0 (521.8, 2070.0)	1160.0 (505.0, 2020.0)	1195.0 (545.0, 2140.0)	0.327

*AKI =* acute kidney injury

### Secondary outcomes with propensity score-matched cohorts

The in-hospital mortality in permissive low-MAP group differed from that in high-MAP group (27.6% (359/1301) vs. 21.8% (283/1301), p < 0.001). Compared with the high-MAP group, the length of ICU-free stay in the permissive low-MAP group is shorter (25.7 (9.6, 28.1) vs. 24.6 (3.8, 27.9) days, p < 0.001). Urine output within the first day after admission to ICU in the permissive low-MAP group was significantly less than that in the high-MAP group (1150.0 (640.0, 1880.0) vs. 1300.0 (665.0, 2170.0) ml, p = 0.007), and the same could be said for the second day after ICU admission (1059.0 (485.0, 1872.0) vs. 1212.0 (579.2, 2026.0) mL, p = 0.013). However, on the third day there was no statistical significance between the two groups. The difference between the permissive low-MAP group and high-MAP group was not significant in the comparison of concomitant AKI within 48 h and 7 days of ICU admission. These detailed results are described in Table 2.

### Sensitivity analysis

**Fig. 3:**
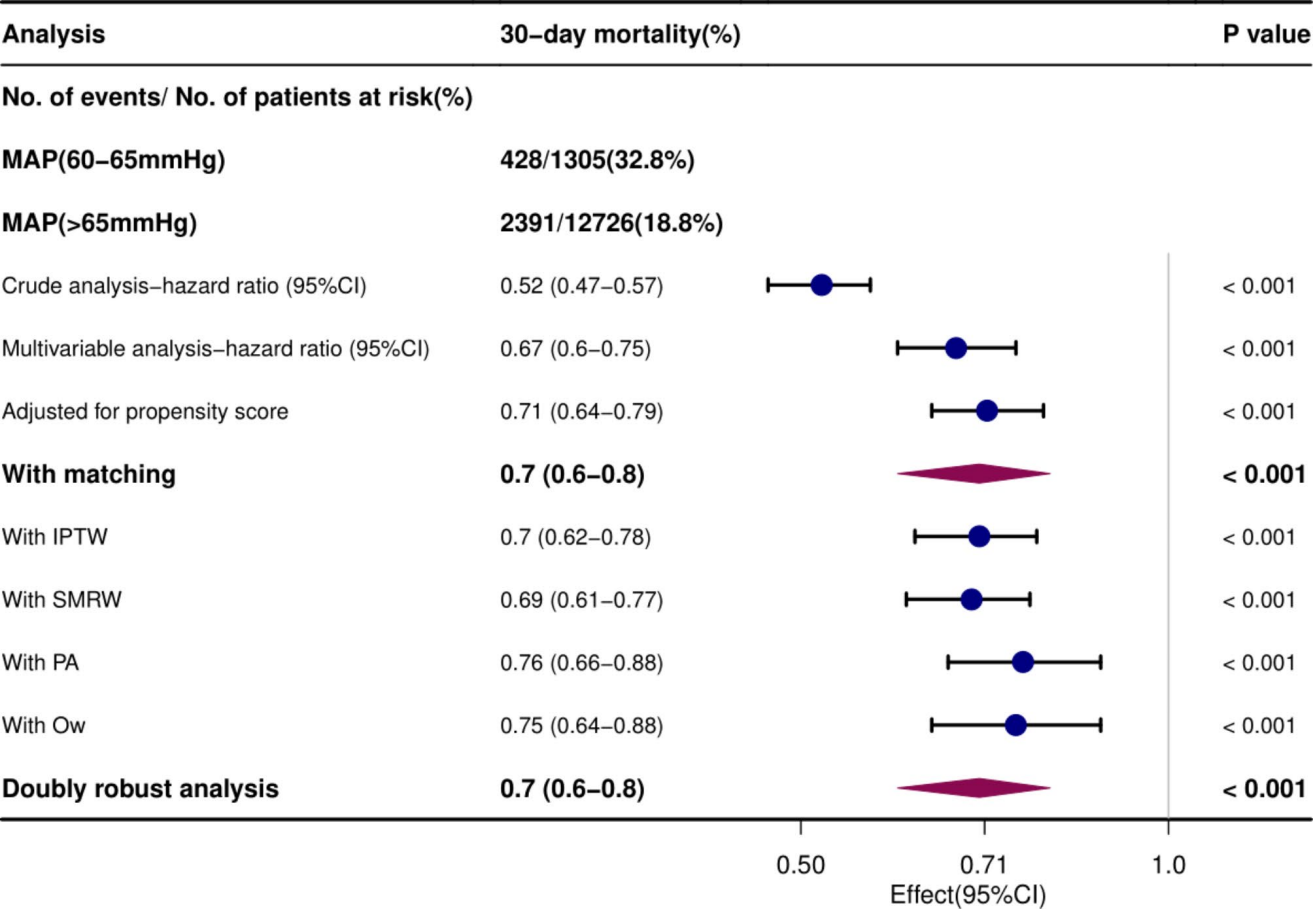
Associations between different MAP levels and the outcome in the crude analysis, multivariable analysis, and propensity-score analyses

In the entire cohort (*n* = 14,031), after adjusting for all covariates in Table 1 by propensity score (HR = 0.71; 95% CI, 0.64–0.79, p < 0.001), the association between the high-MAP group and decreased 30-day mortality was confirmed by COX hazard model multivariate analysis (HR = 0.67; 95% CI, 0.6–0.75, p < 0.001) in sepsis patients. Inverse probability of treatment weighing (IPTW), Standardized mortality ratio weighting (SMRW), PA weighting (PA), Overlap Weighting (OW) and Doubly robust analysis were performed, and HR values remained between 0.69 and 0.76 consistently, with p values less than 0.001 (Fig. 3).

We conducted multiple imputation before propensity score matching, and the result of COX multivariate regression analysis including all covariates in Table 1 identified that high-MAP was associated with reduced short-term mortality. In the adjusted model, the ORs for 30-day, 60-day and 100-day mortality associated with high-MAP were 0.67 (95% CI, 0.6–0.75, p < 0.001), 0.7 (95% CI, 0.63–0.77, p < 0.001), 0.73 (95% CI, 0.66–0.8, p < 0.001), respectively (Table S4). While after PSM, the ORs for 30-day, 60-day and 100-day mortality associated with high-MAP were 0.66 (95% CI, 0.57–0.76, p < 0.001), 0.67 (95% CI, 0.59–0.77, p < 0.001), 0.7 (95% CI, 0.61–0.79, p < 0.001), respectively (Table S5).

## Discussion

In this retrospective cohort study with propensity score matching, we had three major findings and four secondary findings.

Our major finding was that the mortality of 30-days, 60-days, and 100-days in the permissive low-MAP group was obviously higher than that in the high-MAP group. Unexpectedly, the high-MAP was positively associated with decreased short-term mortality. The results remained robust after utilizing PSM, adjustment of confounding variables, IPTW, SMRW, PA, OW and Doubly robust analysis. We also found that sepsis patients in the high-MAP group had lower in-hospital mortality than those in the permissive low-MAP group. Accordingly, the length of ICU-free stay and urine output on days 1 and 2 of ICU admission in the high-MAP group were significantly higher than those in the permissive low MAP group. When referring to the urine output on day 3 and the concomitant AKI within 48 h and 7 days of ICU admission, the high-MAP group had better performance than the permissive low-MAP group, though the differences were not statistically significant.

Our secondary findings related to pressure indices, which require some context before enumerating. Pressure indices are widely used in clinical practice, especially in the monitoring of hemodynamics. Because these indices are more accessible, economical, and can be converted into visual dynamic indicators that can be monitored by clinicians in real time with simple sensors. Over the past decade, there have been many large-scale studies [22–25] exploring the relationship between different pressure indexes and the prognosis of sepsis patients, such as central venous pressure (CVP), pulmonary capillary wedge pressure (PCWP), and MAP. Sepsis patients can develop tissue and organ hypoperfusion early and progress rapidly to multi-organ failure. MAP is a widely-used parameter indicating the driving force of tissue and organ perfusion pressure, and appropriate MAP is prerequisite for adequate perfusion of major organs, so MAP has always been of more concern among critical care researchers [26, 27]. At the same time, it is the only pressure indicator that has been constantly mentioned and recommended in successive international guidelines for the management of sepsis and septic shock [13, 28–32], which highlights its clinical importance. A retrospective study based on big database [33], which included 5347 patients with distributive shock, observed that the mortality of ICU patients with MAP < 80, < 75, < 65, < 60 and < 55 mmHg for less than 2 h were 20.2%, 18.7%, 22.1%, 26.1%, and 31.0%, respectively, which was consistent with the results of our study. And from our study, it is not difficult to find that the high-MAP group had lower 30-day, 60-day, 100-day and in-hospital mortality than the permissive low-MAP group before and after PSM, and the robustness of our results has been validated by IPTW, SMRW, PA, OW and Doubly robust analysis, whereas previous studies did not prove the stability of the outcome by further sensitivity analysis.

Patients with sepsis have a high mortality rate, with clinical symptoms such as oliguria, shortness of breath, intolerance of enteral nutrition, consciousness alteration, and coagulation abnormality in a short time, which can rapidly progress to multiple organ dysfunction syndrome (MODS) if left untreated. All of these are inextricably associated with early hypotension in septic patients. Organ perfusion is distributed nonlinearly when in shock, preferentially supplying vital tissues and organs such as the heart, brain, and kidneys [34, 35], but when blood pressure is low to a certain level the organism will lose the ability to provide protection. For the overall population of sepsis, setting an initial MAP target too low would bring the risks of hypoperfusion of vital organs, however, patients with extremely high preset MAP are more likely to face the danger of re-injury from higher doses of vasoactive drugs. Although there were no significant differences in short-term mortality between the permissive low-MAP group and the high-MAP group in the previous RCT study, it was common that the actual MAP of patients in the permissive low-MAP group was significantly higher than the preset upper limit (65 mmHg). Combined with the results of our observation, we hypothesized the low inflection point of MAP corresponding to short-term mortality in sepsis patients may be located near to the upper side of 65 mmHg. In the future, we hope to perform continuous curve fitting in different databases to identify the best MAP value for sepsis patients so as to provide the best clinical evidence for medical management.

There are, however, two studies that are not totally consistent with the results of the present study. In a multicenter, randomized clinical trial conducted in 65 ICU centers in the UK [11], a total of 2598 patients aged 65 years and older with vasodilatory hypotension were randomized to the permissive hypotension group (60–65 mmHg) and the routine care group. Data of 1221 patients in the permissive hypotension group and 1242 patients in the routine care group were ultimately included to primary outcomes analysis after obtaining delayed retrospective consent. At day 90, 500 (41.0%) patients died in the permissive hypotension group vs. 544 (43.8%) in the routine care group (p = 0.15). In another open-label multicenter RCT involving 11 ICUs in Canada and the United States [12], a total of 120 patients with vasodilatory shock were enrolled and randomized 1:1 into a high MAP group (75–80 mmHg) versus a low MAP group (60–65 mmHg). Clinical data from 60 patients in the low MAP group and 58 patients in the high MAP group were eventually analyzed for the primary outcome after two participants withdrew their informed consent. The proportion of patients who died or suffered from persistent organ dysfunction within 28 days was similar between the two groups (44% in the low MAP group vs. 46% in the high MAP group, p = 0.21). In a study of 65 centers in the UK [11], they observed that the actual MAP in the permissive hypotension group had reached 66.7 mmHg, higher than the upper limit of the preset MAP values. The participants of the low MAP group from 11 ICUs in Canada and the United States [12] also had actual MAP significantly higher than the preset upper limit, which may account for the inconsistency between the results of our study and these two studies.

Another finding of our team, also based on the large database [36], that performed adjustment for confounders and curve fitting found that the MAP corresponding to the lowest 30-day mortality in sepsis patients may be around 68.6 mmHg. Further analysis showed that the 30-day mortality decreased with an increase of MAP for those whose MAP was less than 68.6 mmHg, and the association between 30-day mortality and MAP changed to become statistically insignificant when MAP was higher than 68.6 mmHg. The fact that the 30-day mortality was not statistically different between the permissive low MAP group and the high MAP group in the 65-center study in the United Kingdom [11] and the study involving 11 ICUs in Canada and the United States [12] may be due to the fact that the actual blood pressure in the permissive hypotension group was very close to the low inflection point between MAP and 30-day mortality.

Our study has several advantages over other studies. Based on the large MIMIC-III public database, our study benefited from a large sample size and information disclosure, which helps avoid the effect of small sample studies on the stability of results due to individual differences or extreme values [37]. Secondly, the data of MIMIC-III database are all generated during daily consultations, which are divided according to established facts before the study design, perfectly solving the dilemma that the actual blood pressure cannot be maintained at a preset level in RCT studies [38, 39].

There are also some limitations to this study. Firstly, this is a retrospective cohort study, so it cannot have strict inclusion and exclusion criteria like RCT studies, where covariates can be purposefully collected at the beginning of the study. Hence, all relevant covariates are somewhat incomplete. In addition, due to the intrinsic limitation of retrospective study, the observed association between average MAP and mortality would be hard to indicate causality. Secondly, many patients in this study had multiple comorbidities, and these patients could not be excluded at the time of enrollment as in an RCT study. However, this study is much closer to the actual situation of patients and is highly generalizable with better extrapolation by enrolling patients consecutively regardless of the disease severity [40]. Besides, we used multiple imputation to minimize bias due to missing data and used PSM to equalize the two groups of patients. But it is a pity that the adjustment for time-dependent covariates is difficult to perform in PSM study due to the difficulty of matching. In addition, despite the large scale of our sample, it is still a single-center study and lacks patients from different countries and ethnicities to offset the differences between regions and ethnic groups. Finally, the accuracy of blood pressure values in the MIMIC-III database may be affected by diverse blood pressure monitoring locations and different types of sphygmomanometers, so blood pressure measurements are not as strictly standardized as those in RCT studies.

In addition, it is noted that the optimal initial MAP setting for sepsis patients may be different among different subgroups, and there is no recommendation for initial MAP of different subgroups of sepsis patients in the current guidelines. We hope to expand the sample size in the future to further investigate the relationship between various pressure indicators and the prognosis of sepsis patients. We also plan to continue exploring the optimal initial MAP setting of sepsis patients among different subgroups using multiple clinical large databases with the aim to provide a foundation for individualized protocols for sepsis patients among different subgroups, which will gradually unveil the association between different pressure indicators and various poor prognoses of sepsis patients.

## Conclusion

In summary, our study indicated that permissive low-MAP management did not achieve a similar prognosis as high-MAP management, and the 30-day, 60-day, 100-day and in-hospital mortalities were lower in patients in the high-MAP group than in the permissive low-MAP group. The results of the length of ICU-free stay and urinary output within the first two days of ICU admission in the high-MAP group were also positive. Therefore, when it comes to the management of septic patients in a population-based protocol, we do not recommend an overly conservative MAP management protocol (permissive low-MAP management) based on our study.

## Electronic supplementary material

Below is the link to the electronic supplementary material.

Supplementary Material 1

Supplementary Material 2

Supplementary Material 3

Supplementary Material 4

Supplementary Material 5

Supplementary Material 6

Supplementary Material 7

Supplementary Material 8

Supplementary Material 9

Supplementary Material 10

Supplementary Material 11

Supplementary Material 12

## Data Availability

The datasets generated and/or analysed during the current study are available in the Physinot repository(mimic.physionet.org ), but restrictions apply to the availability of these data, which were used under license(certification number: 40,489,150) for the current study, and so are not publicly available except from the corresponding author (Dr.Qing He) upon reasonable request and with permission of the holder of the database.

## Abbreviations

MAP: Mean arterial pressure
RCT: Randomized controlled study
MIMIC: Medical Information Mart for Intensive Care
PSM: Propensity score matching; IPTW inverse probability of treatment weighing
SMRW: Standardized mortality ratio weighting
OW: Overlap Weighting
ICU: Intensive care unit
AIDS: Acquired immune deficiency syndrome
RECORD: Reporting of Studies Conducted using observation Routinely Collected Health Data
IRB: Institutional Review Board
SQL: Structured query language
IQR: Interquartile range
SMD: Standardized mean differences
AKI: Acute kidney injury
CVP: Central venous pressure
PCWP: Pulmonary capillary wedge pressure
MODS: Multiple organ dysfunction syndrome
MICU: Medical intensive care unit
SICU: Surgical intensive care unit
TSICU: Trauma surgical intensive care unit
CCU: Coronary care unit
CSRU: Cardiac surgery unit
Sofa: Sequential Organ Failure Assessment
SAPS II: Simplified Acute Physiology Score II
OASIS: Oxford Acute Severity of Illness Score
MV: Mechanical ventilation
RRT: Renal replacement therapy
CHF: Congestive heart failure
COPD: Chronic obstructive pulmonary disease
WBC: White blood cell
PO2: Partial pressure of oxygen
PCO2: Partial pressure of carbon dioxide
Lac: Lactic acid
