# Supplementary figures and images for: Association between different MAP levels and 30-day mortality in sepsis patients: a propensity-score-matched, retrospective cohort study
Source: BMC Anesthesiol. 2023 Apr 6;23:116. doi: 10.1186/s12871-023-02047-7 (PMC10077659; doi:10.1186/s12871-023-02047-7)

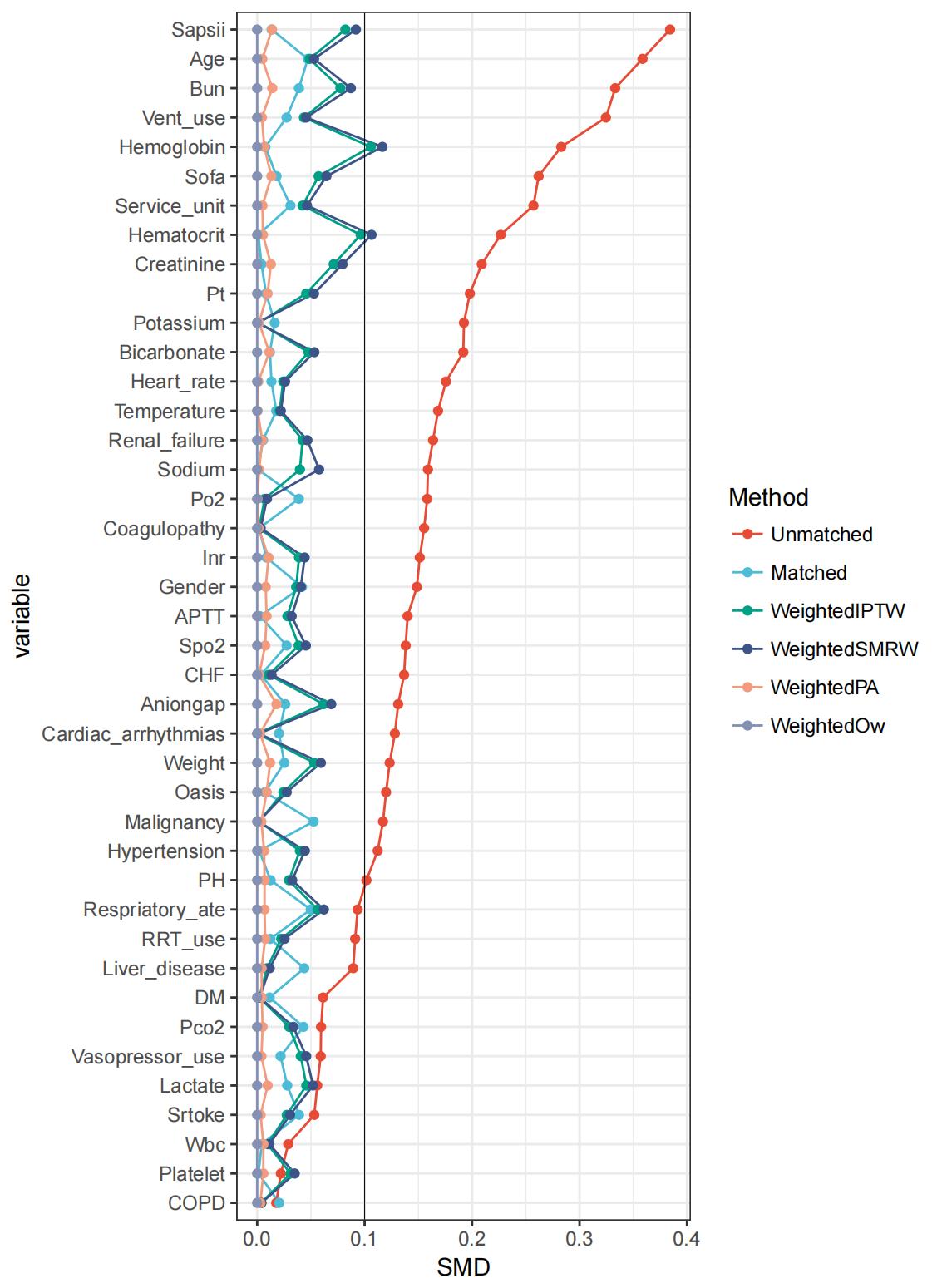

Supplement: Supplementary file 1 — Supplementary Material 1 [file 12871_2023_2047_MOESM1_ESM.jpg]

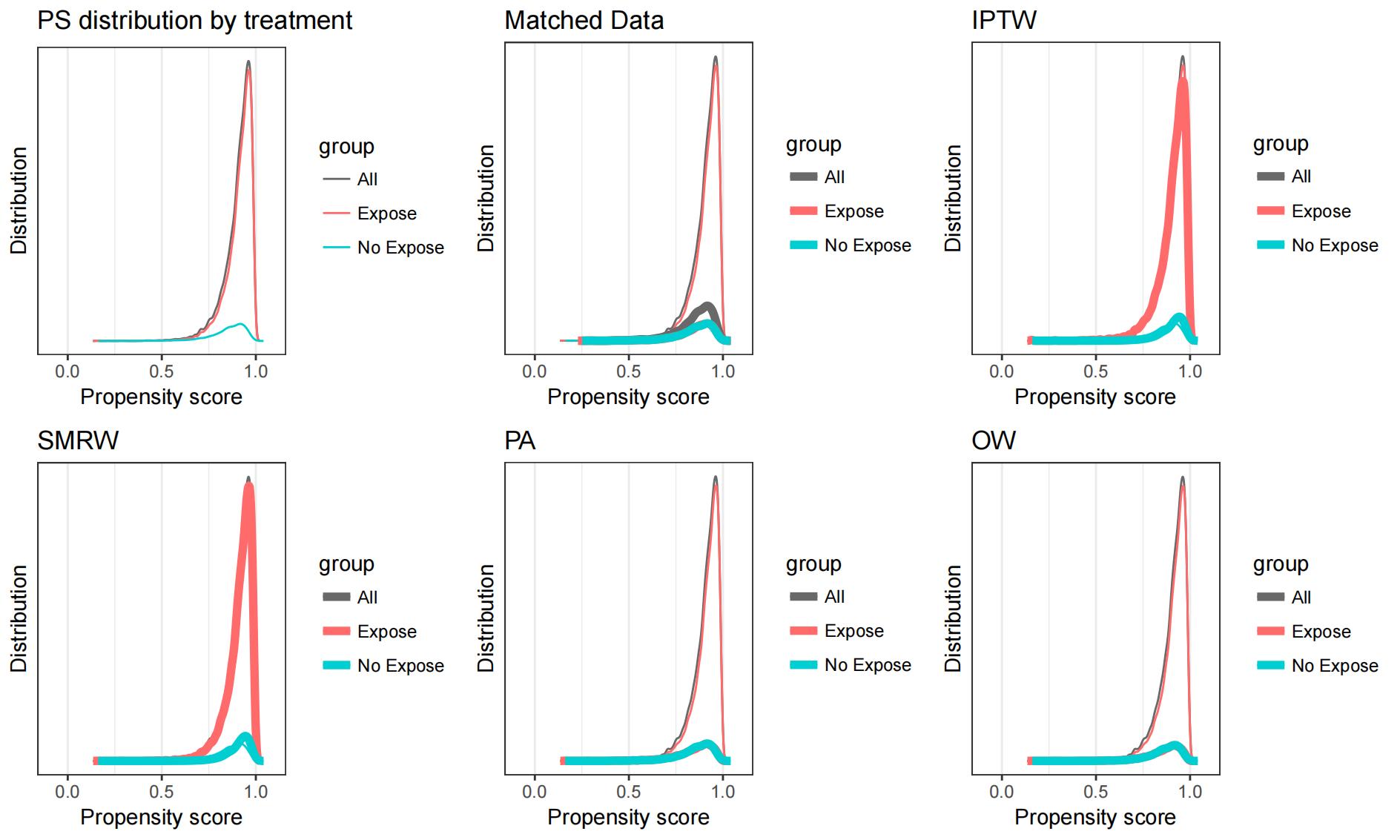

Supplement: Supplementary file 2 — Supplementary Material 2 [file 12871_2023_2047_MOESM2_ESM.jpg]

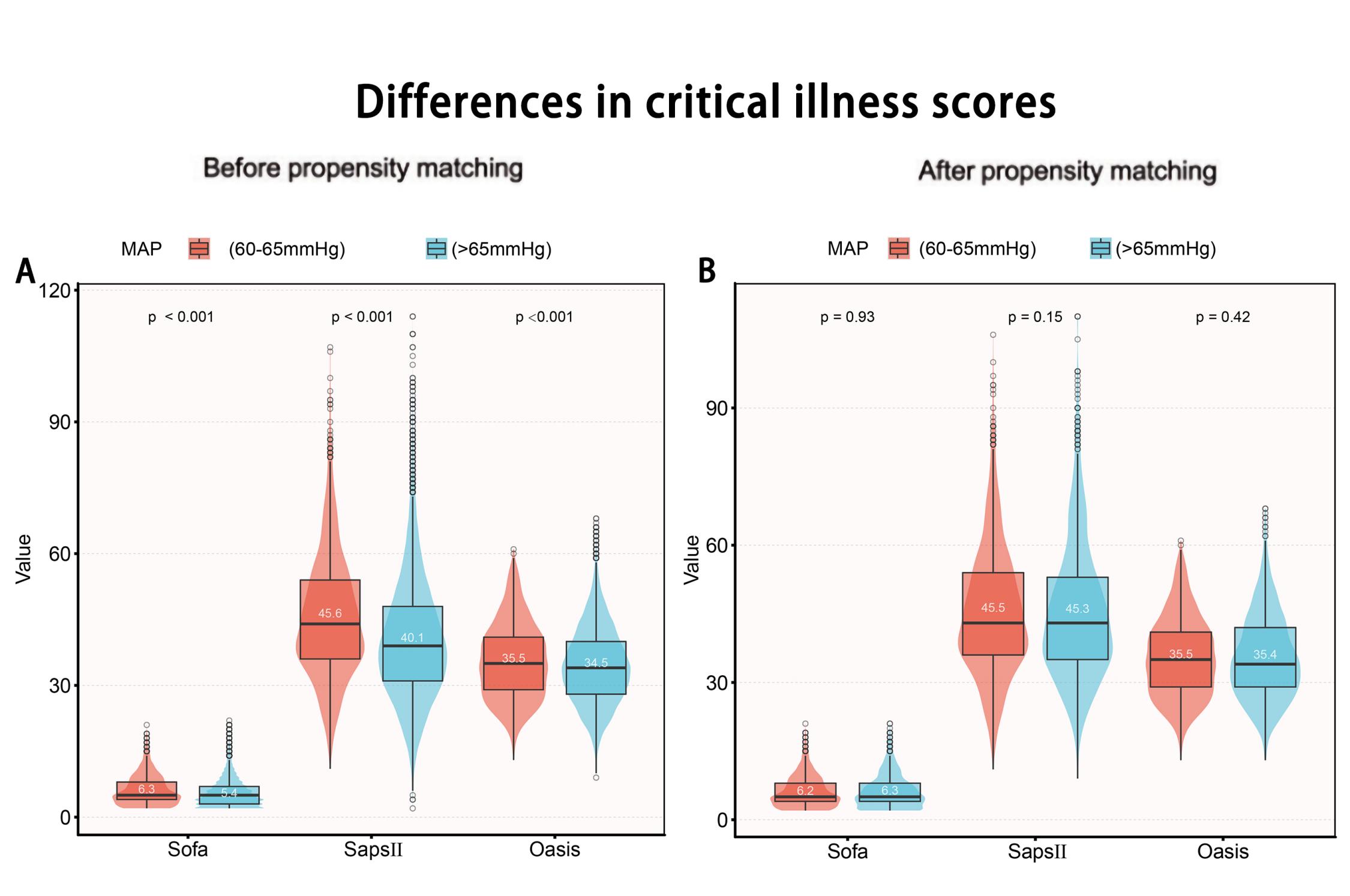

Supplement: Supplementary file 3 — Supplementary Material 3 [file 12871_2023_2047_MOESM3_ESM.jpg]

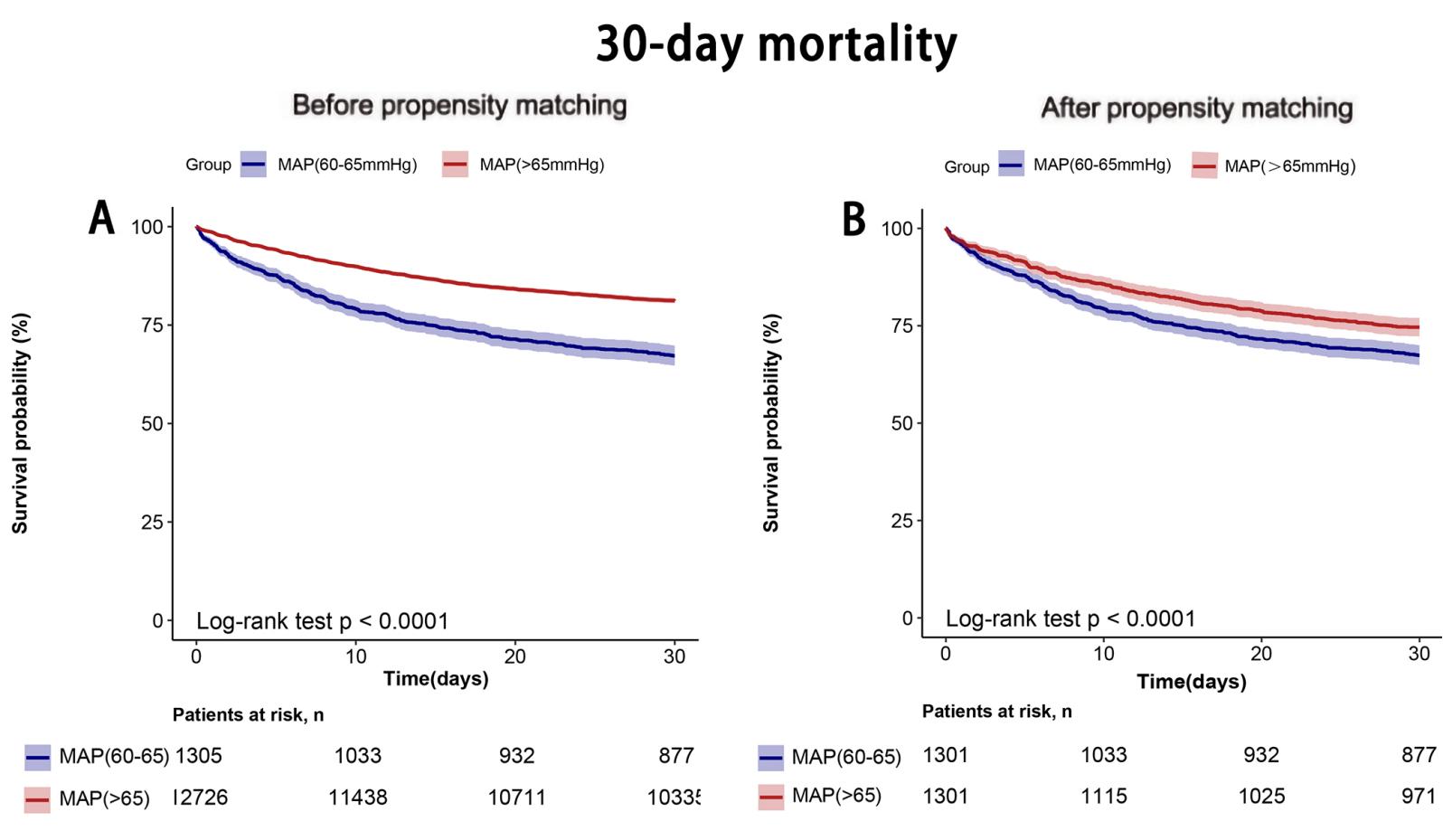

Supplement: Supplementary file 4 — Supplementary Material 4 [file 12871_2023_2047_MOESM4_ESM.jpg]

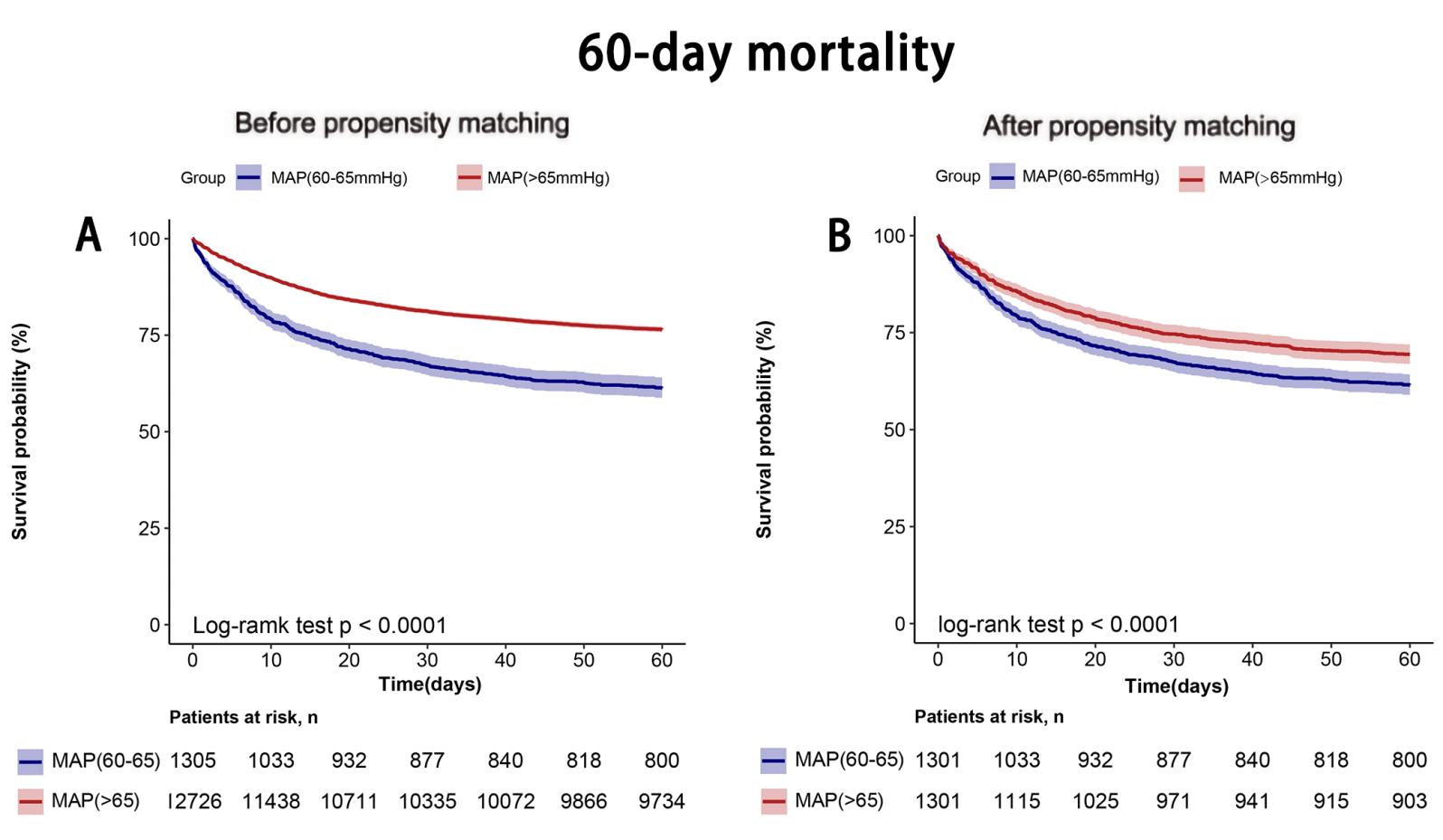

Supplement: Supplementary file 5 — Supplementary Material 5 [file 12871_2023_2047_MOESM5_ESM.jpg]

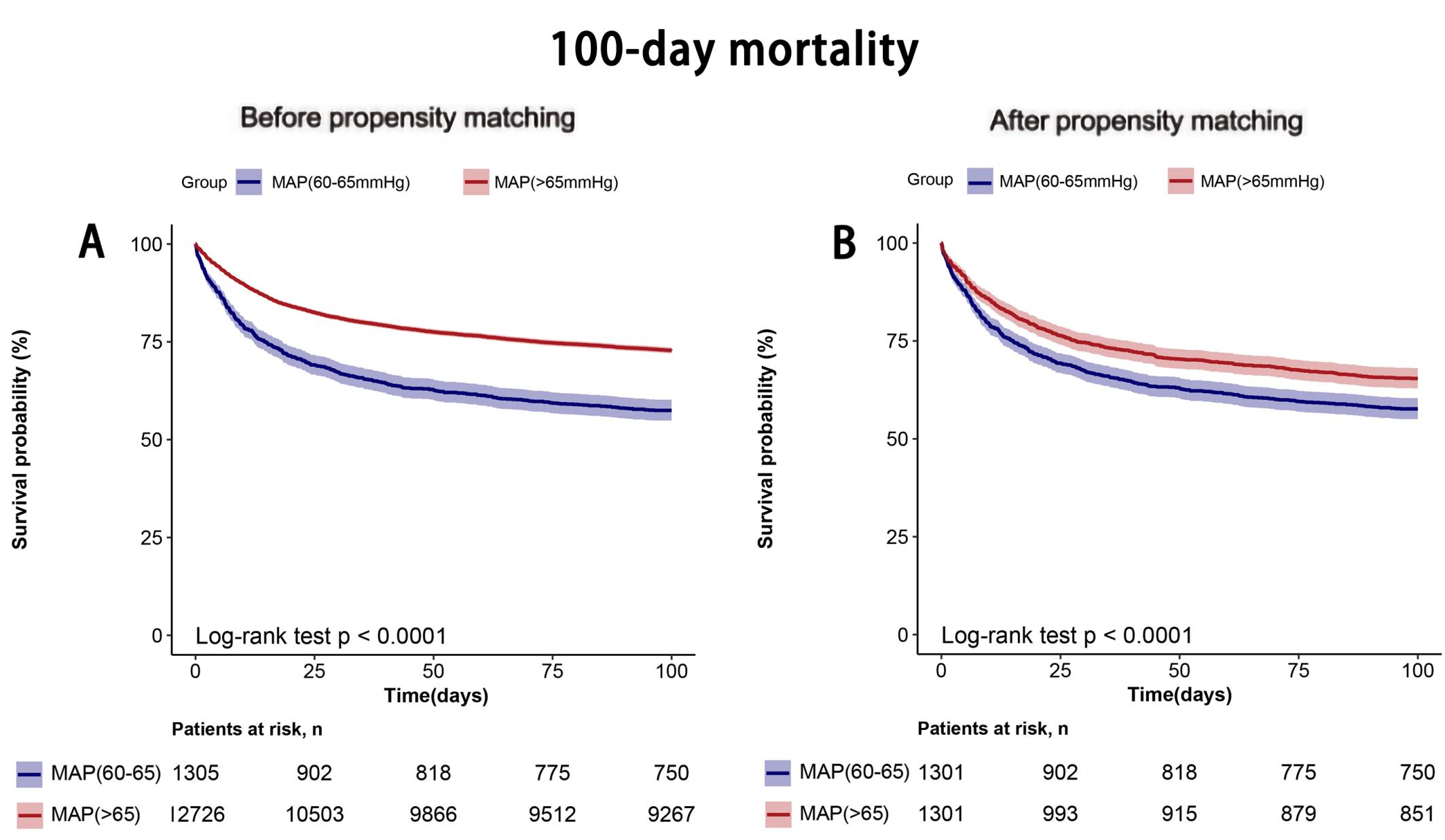

Supplement: Supplementary file 6 — Supplementary Material 6 [file 12871_2023_2047_MOESM6_ESM.jpg]
