# Supplementary material for: Association between different MAP levels and 30-day mortality in sepsis patients: a propensity-score-matched, retrospective cohort study
Source: BMC Anesthesiol. 2023 Apr 6;23:116. doi: 10.1186/s12871-023-02047-7 (PMC10077659; doi:10.1186/s12871-023-02047-7)
Supplement: Supplementary file 7 — Supplementary Material 7 [file 12871_2023_2047_MOESM7_ESM.docx]

| **Table S1:** Percentage of missing data in the variables of interest | | |
| --- | --- | --- |
|  |  | MIMIC-III (n=14607) |
|  | Miss frequencies | Miss percentage % |
| Participants | 0 |  |
| Age | 0 | 0.00% |
| Sex, n (%) | 0 | 0.00% |
| female | 0 | 0.00% |
| male | 0 | 0.00% |
| Weight, | 388 | 2.77% |
| Service.unit, n (%) |  |  |
| MICU | 0 | 0.00% |
| SICU/TSICU | 0 | 0.00% |
| CCU/CSRU | 0 | 0.00% |
| Severity of illness |  |  |
| SOFA | 0 | 0.00% |
| SAPS Ⅱ | 0 | 0.00% |
| OASIS | 0 | 0.00% |
| Vital signs |  |  |
| Heart rate | 1 | 0.01% |
| mean BP | 0 | 0.00% |
| Respiratory rate | 14 | 0.10% |
| Temperature | 193 | 1.38% |
| SpO_2_ | 9 | 0.06% |
| ELS |  |  |
| CRRT use, n (%) | 0 | 0.00% |
| MV use, n (%) | 0 | 0.00% |
| Vasopressor use,n(%) | 61 | 0.43% |
| Comorbidities, n (%) |  |  |
| CHF | 0 | 0.00% |
| Cardiac arrhythmias | 0 | 0.00% |
| Hypertension | 0 | 0.00% |
| Stroke | 0 | 0.00% |
| COPD | 0 | 0.00% |
| Diabetes mellitus | 0 | 0.00% |
| Renal failure | 0 | 0.00% |
| Liver disease | 0 | 0.00% |
| Malignancy | 0 | 0.00% |
| Coagulopathy | 0 | 0.00% |
| Laboratory tests |  |  |
| WBC | 71 | 0.51% |
| Hemoglobin | 55 | 0.39% |
| Platelet | 61 | 0.43% |
| Hematocrit | 38 | 0.27% |
| Inr | 1200 | 8.55% |
| PT | 1200 | 8.55% |
| APTT | 1230 | 8.77% |
| BUN | 36 | 0.26% |
| Creatinine | 35 | 0.25% |
| Sodium | 30 | 0.21% |
| Potassium | 26 | 0.19% |
| Bicarbonate | 58 | 0.41% |
| pH | 3272 | 23.32% |
| PO_2_ | 3594 | 25.61% |
| PCO_2_ | 3593 | 25.61% |
| Lac | 3993 | 28.46% |
| Anion gap | 168 | 1.20% |
| *MICU* medical intensive care, *SICU* surgical intensive care unit, *TSICU* trauma surgical intensive care unit, *CCU* coronary care unit, *CSRU* cardiac surgery unit, *SOFA* Sequential Organ Failure Assessment, *SAPS II* Simplified Acute Physiology Score II, *OASIS* Oxford Acute Severity of Illness Score, *ELS* extracorporeal life support, *MV* mechanical ventilation, *RRT* renal replacement therapy, *CHF* congestive heart failure, *COPD* chronic obstructive pulmonary disease, *MAP* mean arterial pressure, *WBC* white blood cell, *PT* prothrombin time, *APTT* activated partial thromboplastin time, *BUN* blood urea nitrogen, *PO_2_* partial pressure of oxygen, *PCO_2_* partial pressure of carbon dioxide, *Lac* lactic acid | | |
