# Supplementary material for: Association between different MAP levels and 30-day mortality in sepsis patients: a propensity-score-matched, retrospective cohort study
Source: BMC Anesthesiol. 2023 Apr 6;23:116. doi: 10.1186/s12871-023-02047-7 (PMC10077659; doi:10.1186/s12871-023-02047-7)
Supplement: Supplementary file 8 — Supplementary Material 8 [file 12871_2023_2047_MOESM8_ESM.docx]

| **Table S2:** One-way ANOVA of covariates and outcomes | | |
| --- | --- | --- |
|  | HR(95%CI) | P value |
|  |  |  |
| Male | 1.0015 (0.93,1.0785) | 0.968 |
| Age | 1.02 (1.02,1.02) | < 0.001 |
| Service unit, n (%) |  |  |
| MICU | 1(Reference) |  |
| SICU/TSICU | 0.6 (0.55,0.66) | < 0.001 |
| CCU/CSRU | 0.58 (0.52,0.64) | < 0.001 |
| Weight | 0.9906 (0.9889,0.9924) | < 0.001 |
| Vital signs |  |  |
| Heart rate | 1.01 (1.01,1.02) | < 0.001 |
| Respiratory rate | 1.08 (1.07,1.09) | < 0.001 |
| Temperature | 0.67 (0.63,0.71) | < 0.001 |
| SpO_2_ | 0.92 (0.91,0.92) | < 0.001 |
| Severity of illness |  |  |
| SOFA | 1.19 (1.18,1.2) | < 0.001 |
| SAPS Ⅱ | 1.05 (1.05,1.06) | < 0.001 |
| OASIS | 1.08 (1.07,1.08) | < 0.001 |
| Comorbidities, n (%) |  |  |
| CHF | 1.48 (1.37,1.6) | < 0.001 |
| Cardiac arrhythmias | 1.49 (1.38,1.61) | < 0.001 |
| Hypertension | 1.02 (0.92,1.13) | 0.681 |
| Stroke | 0.76 (0.61,0.94) | 0.013 |
| COPD | 1.04 (0.96,1.14) | 0.332 |
| Diabetes mellitus | 0.88 (0.81,0.95) | 0.002 |
| Renal failure | 1.11 (1.01,1.22) | 0.028 |
| Liver disease | 1.35 (1.19,1.52) | < 0.001 |
| Malignancy | 2.36 (2.15,2.59) | < 0.001 |
| Coagulopathy | 1.72 (1.58,1.87) | < 0.001 |
| ELS, n (%) |  |  |
| RRT use | 1.32 (1.22,1.42) | < 0.001 |
| MV use | 1.39 (1.2,1.62) | < 0.001 |
| Vasopressor use | 1.76 (1.62,1.92) | < 0.001 |
| Laboratory tests |  |  |
| WBC | 1.0039 (1.0029,1.0049) | < 0.001 |
| Hemoglobin | 0.96 (0.95,0.98) | < 0.001 |
| Platelet | 0.9993 (0.999,0.9996) | < 0.001 |
| Hematocrit | 0.9983 (0.9922,1.0045) | 0.596 |
| Inr | 1.08 (1.07,1.09) | < 0.001 |
| PT | 1.01 (1.01,1.02) | < 0.001 |
| APTT | 1.0056 (1.0046,1.0067) | < 0.001 |
| Bun | 1.01 (1.01,1.01) | < 0.001 |
| Creatinine | 1.06 (1.04,1.07) | < 0.001 |
| Potassium | 1.42 (1.33,1.51) | < 0.001 |
| Sodium | 0.999 (0.9924,1.0057) | 0.774 |
| Bicarbonate | 0.95 (0.94,0.95) | < 0.001 |
| pH | 0.1 (0.07,0.15) | < 0.001 |
| PO_2_ | 0.998 (0.9976,0.9984) | < 0.001 |
| PCO_2_ | 0.9981 (0.9946,1.0015) | 0.269 |
| Lac | 1.13 (1.12,1.14) | < 0.001 |
| Anion gap | 1.07 (1.06,1.08) | < 0.001 |
| *MICU* medical intensive care, *SICU* surgical intensive care unit, *TSICU* trauma surgical intensive care unit, *CCU* coronary care unit, *CSRU* cardiac surgery unit, *SOFA* Sequential Organ Failure Assessment, *SAPS II* Simplified Acute Physiology Score II, *Oasis* Oxford Acute Severity of Illness Score, *ELS* extracorporeal life support, *MV* mechanical ventilation, *RRT* renal replacement therapy, *CHF* congestive heart failure, *COPD* chronic obstructive pulmonary disease, *MAP* mean arterial pressure, *WBC* white blood cell, *WBC* white blood cell, *PT* prothrombin time, *APTT* activated partial thromboplastin time, *BUN* blood urea nitrogen, *PO_2_* partial pressure of oxygen, *PCO_2_* partial pressure of carbon dioxide, *Lac* lactic acid | | |
|  |  |  |
