# Supplementary material for: Association between different MAP levels and 30-day mortality in sepsis patients: a propensity-score-matched, retrospective cohort study
Source: BMC Anesthesiol. 2023 Apr 6;23:116. doi: 10.1186/s12871-023-02047-7 (PMC10077659; doi:10.1186/s12871-023-02047-7)
Supplement: Supplementary file 9 — Supplementary Material 9 [file 12871_2023_2047_MOESM9_ESM.docx]

| **Table S3:** The distribution of the MAP | | | | | | | | |
| --- | --- | --- | --- | --- | --- | --- | --- | --- |
| Characteristic |  | Original cohort |  |  |  | Matched cohort |  |  |
|  |  | MAP(60-65 mmHg) | MAP(>65 mmHg) | p |  | MAP(60-65 mmHg) | MAP(>65 mmHg) | p |
|  |  | (n = 1301) | (n = 1301) |  |  | (n = 1301) | (n = 1301) |  |
| MAP (mmHg), Mean ± SD |  | 62.9 ± 1.4 | 78.6 ± 9.7 | < 0.001 |  | 62.9 ± 1.4 | 76.6 ± 9.3 | < 0.001 |
| MAP(mmHg), Median (IQR) |  | 63.0 (61.7, 64.1) | 76.7 (71.2, 83.8) | < 0.001 |  | 63.0 (61.7, 64.1) | 74.5 (69.7, 81.2) | < 0.001 |
