# Supplementary material for: Association between different MAP levels and 30-day mortality in sepsis patients: a propensity-score-matched, retrospective cohort study
Source: BMC Anesthesiol. 2023 Apr 6;23:116. doi: 10.1186/s12871-023-02047-7 (PMC10077659; doi:10.1186/s12871-023-02047-7)
Supplement: Supplementary file 10 — Supplementary Material 10 [file 12871_2023_2047_MOESM10_ESM.docx]

**Table S4**: Relationship between pre-match study and short-term death

| Exposure | Nonadjusted | | adjusted | |
| --- | --- | --- | --- | --- |
|  | HR(95%CI) | *P* value | HR(95%CI) | *P* value |
| MAP:(>65 mmHg) vs (60-65 mmHg) |  |  |  |  |
| 30-day mortality | 0.52 (0.47~0.57) | <0.001 | 0.67 (0.6~0.75) | <0.001 |
| 60-day mortality | 0.54 (0.49~0.59) | <0.001 | 0.7 (0.63~0.77) | <0.001 |
| 100-day mortality | 0.56 (0.51~0.61) | <0.001 | 0.73 (0.66~0.8) | <0.001 |
