# Supplementary material for: Association between different MAP levels and 30-day mortality in sepsis patients: a propensity-score-matched, retrospective cohort study
Source: BMC Anesthesiol. 2023 Apr 6;23:116. doi: 10.1186/s12871-023-02047-7 (PMC10077659; doi:10.1186/s12871-023-02047-7)
Supplement: Supplementary file 11 — Supplementary Material 11 [file 12871_2023_2047_MOESM11_ESM.docx]

**TableS5**: Relationship between post-match and short-term death

| Exposure | Nonadjusted | | adjust | |
| --- | --- | --- | --- | --- |
|  | HR(95%CI) | *P* value | HR(95%CI) | *P* value |
| MAP:(>65 mmHg) vs (60-65 mmHg) |  |  |  |  |
| 30-day mortality | 0.74 (0.64~0.85) | <0.001 | 0.66 (0.57~0.76) | <0.001 |
| 60-day mortality | 0.75 (0.65~0.85) | <0.001 | 0.67 (0.59~0.77) | <0.001 |
| 100-day mortality | 0.76 (0.67~0.86) | <0.001 | 0.7 (0.61~0.79) | <0.001 |
