# Supplementary material for: Association between different MAP levels and 30-day mortality in sepsis patients: a propensity-score-matched, retrospective cohort study
Source: BMC Anesthesiol. 2023 Apr 6;23:116. doi: 10.1186/s12871-023-02047-7 (PMC10077659; doi:10.1186/s12871-023-02047-7)
Supplement: Supplementary file 12 — Supplementary Material 12 [file 12871_2023_2047_MOESM12_ESM.docx]

Additional files：

Figure S1: Plot of SMD after Propensity score analysis. Figure S2: Density Plot of Propensity score in different methods. Figure S3: Differences in critical illness scores. Figure S4: Kaplan-Meier Survival Curves for day 30 of sepsis patients. Figure S5: Kaplan-Meier Survival Curves for day 60 of sepsis patients. Figure S6: Kaplan-Meier Survival Curves for day 100 of sepsis patients. Table S1: Percentage of missing data in the variables of interest. Table S2: One-way ANOVA of covariates and outcomes. Table S3: The distribution of the MAP. Table S4: Relationship between pre-match study and short-term death. Table S5: Relationship between post-match study and short-term death.
